# Three new *Melanoleuca* species (Agaricales, Basidiomycota) from north-eastern China, supported by morphological and molecular data

**DOI:** 10.3897/mycokeys.80.64369

**Published:** 2021-06-03

**Authors:** Ying Pei, Hong-Bo Guo, Tie-Zhi Liu, Wei-Qiang Qin, Di Zhao, Xiao-Jian Qi, Xiao-Dan Yu

**Affiliations:** 1 College of Biological Science and Technology, Shenyang Agricultural University, Shenyang 110866, China Shenyang Agricultural University Shenyang China; 2 College of Life Engineering, Shenyang Institute of Technology, Fushun 113122, China Shenyang Institute of Technology Fushun China; 3 College of Chemistry and Life Sciences, Chifeng University, Chifeng 024000, China Chifeng University Chifeng China; 4 Jishou University, Zhangjiajie 42700, China Jishou University Zhangjiajie China

**Keywords:** Agaricales, morphology, phylogenetic analysis, Pluteoid clade, taxonomy

## Abstract

Three new *Melanoleuca* species, *M. chifengense*, *M. griseoflava* and *M. pallidorosea*, were discovered in the northeast of China. *Melanoleuca chifengense* is morphologically characterised by its grey to yellowish-grey pileus, decurrent lamellae, grey to yellowish-brown stipe, yellowish-grey context, ellipsoid basidiospores with irregular warts and lack of hymenial cystidia. *Melanoleuca griseoflava* is mainly characterised by its greyish-brown pileus, adnexed to adnate lamellae, greyish-yellow context, fusiform cystidia and almost reticulate basidiospores. *Melanoleuca pallidorosea* is characterised by its pinkish-white pileus, white and decurrent lamellae, ellipsoid basidiospores with round and scattered warts and lack of hymenial cystidia. The phylogenetic relationship of the three species was determined by the analyses of the ITS region and the combined data matrix (ITS-nrLSU-RPB2), respectively. The results showed that the three species formed three independent lineages. Based on the combination of both morphological and molecular data, *M. chifengense*, *M. griseoflava* and *M. pallidorosea* were confirmed to be new species. The morphological similarities of the three new species is also discussed.

## Introduction

*Melaleuca* Pat. was erected by Patouillard in 1887. As the name ‘*Melaleuca*’ was found to be the same as that of a plant species, Patouillard (1897) changed it to the current name *Melanoleuca* Pat. The genus was traditionally included in the family Tricholomataceae, subtribus Leucopaxillaceae Singer mainly because the species present a regular hymenophoral trama, amyloid basidiospores and a white spore print (Singer 1948; Singer 1986). However, molecular data showed that the genus *Melanoleuca* is close to the species of Pluteaceae and Amanitaceae (Moncalvo et al. 2002; Bodensteiner et al. 2004; Matheny et al. 2006; Garnica et al. 2007; Justo et al. 2011; Vizzini et al. 2011; Binder et al. 2014; Yu et al. 2014). Therefore, *Melanoleuca* was assumed to belong to the Pluteoid clade by Matheny et al. (2006) and Sánchez-García et al. (2014).

The species of *Melanoleuca* are often characterised by having a convex to slightly depressed pileus, mostly hymenial cystidia, amyloid ornamented basidiospores and all hyphae without clamp connections (Singer 1986; Boekhout 1988; Vizzini et al. 2011). The genus *Melanoleuca* always grows directly on humus-rich soil, in meadows, in and outside of woods and is distributed in temperate and frigid zones of both hemispheres (Singer 1986). In recent years, many new species of *Melanoleuca* have been reported around the world (Vizzini et al. 2010, 2011; Sánchez-García et al. 2013; Antonín et al. 2014, 2017; Yu et al. 2014; Nawaz et al. 2017; Xu et al. 2019; Antonín et al. 2021). Up to now, there are 221 validly published names reported in the world (Index Fungorum 2021).

Although *Melanoleuca* has been proved to be a monophyletic group, the classification system within the genus remains controversial. Based on the colour of the pileus and the size of the carpophore, Singer (1986) divided the genus into four sections, i.e. sect. Alboflavidae Singer, sect. Humiles Singer, sect. Oreinae Singer and sect. Melanoleuca Pat. As Boekhout (1988) believed that the cystidia should play an important role in the classification system of *Melanoleuca*, the genus was, therefore, divided into three subgenera, based on the types of cystidia, i.e. subgen. Macrocystis Boekhout, subgen. Melanoleuca Pat. and subgen. Urticocystis Boekhout. Subgen. Macrocystis and subgen. Urticocystis are characterised by the presence of fusiform to lageniform cystidia and urticiform cystidia, respectively while subgen. Melanoleuca is characterised by the absence of cystidia. However, these morphological classification systems are not supported by molecular data. The result of ITS region analysis supported the fact that Melanoleuca included two subgenera, i.e. subgen. Urticocystis and subgen. Melanoleuca (Vizzini et al. 2011). The species of subgen. Melanoleuca are characterised by basidiomata with non-septate macrocystidia. subgen. Urticocystis was composed of the taxa mainly with urticocystidia, but also without any cystidia and with macrocystidia and brightly coloured pilei (Vizzini et al. 2011).

In this paper, the authors studied three *Melanoleuca* species collected in north-eastern China from 2017 to 2019. Morphological observation and phylogenetic analyses confirmed that they are novel taxa in the genus *Melanoleuca*.

## Materials and methods

### Morphological studies

All of the fungal specimens were described and photographed in the field. Specimens were dried in an electric drier and deposited in the Fungal Herbarium of Shenyang Agricultural University (SYAU-FUNGI) and Fungal Herbarium of Chifeng University (CFSZ). Tissue blocks were removed from the inner part of the fresh basidiomata for DNA analyses. Macroscopic characters of the basidiomata described here were based on observations of fresh specimens. The names of colours were based on Kornerup and Wanscher (1963). Methods used for morphological descriptions followed those of Li et al. (2017). For the microscopic study, dried materials were observed in 5% potassium hydroxide (KOH) solution. Melzer’s reagent was used for testing colour reactions of the tissues and basidiospores. The notation “(n/m/p)” of basidiospores indicates that the measurements were conducted for n basidiospores from m basidiomata of p collections. The Q value (length:breadth ratio) for each spore was calculated and the mean values are presented in the descriptions. For observation of the surface of the spores, the gills were covered with a thin gold film by using an Ion Sputter Coater (MC1000, Hitachi, Japan) before imaging by a scanning electron microscope (REGLUS 8100, HITACHI, Japan). Line drawings were prepared by freehand.

### DNA extraction, PCR amplification and sequencing

Total genomic DNA was extracted from fresh blocks of tissue, dried with silica gel using the cetyltrimethylammonium bromide (CTAB) method (Doyle and Doyle 1987). Primer pairs ITS5/ITS4 (White et al. 1990), LR0R/LR5 (Michot et al. 1984) and b6F/b7.1R (Matheny et al. 2007) were used to amplify the internal transcribed spacer (ITS) region, the large subunit nuclear ribosomal RNA (nrLSU) region and the second largest subunit of the nuclear RNA polymerase enzyme II (RPB2), respectively. PCR protocol and sequencing were conducted as described by Wang et al. (2019).

### Phylogenetic analyses

High-quality and representative sequences of *Melanoleuca* in previous studies (Sánchez-García et al. 2013; Yu et al. 2014; Antonín et al. 2014, 2015, 2017; Nawaz et al. 2017; Xu et al. 2019; Antonín et al. 2021) were downloaded from GenBank and aligned with the sequences obtained from this study by Bioedit v7.0.9 (Hall 1999) and MAFFT v7.313 (Katoh and Standley 2013). *Pluteus romellii* (AY854065 for ITS; AY634279 for nrLSU; AY786063 for RPB2) was used as the outgroup in this study. Data partition homogeneity tests (Farris et al. 1995) were implemented in PAUP 4.0b4a (Swofford 2003). This test detected no conflicts among ITS, nrLSU and RPB2 regions (P-value = 0.33), suggesting that sequences of the three genes can be combined for phylogenetic analysis. The final ITS data matrices consisted of 125 samples of 669 characters, whereas the combined data set (ITS-nrLSU-RPB2) consisted of 67 samples of 2204 characters. Maximum likelihood (ML) analysis was performed with RAxML-8.2.10-WIN using a GTR-GAMMA model of evolution (Stamatakis 2014). Nodal bootstrap support (BS) was assessed with nonparametric bootstrapping using 1000 replicates. Bayesian Inference (BI) analysis was conducted with MrBayes v.3.2.6 (Ronquist et al. 2012). ModelFinder (Kalyaanamoorthy et al. 2017) and PartitionFinder 2 (Lanfear et al. 2016) were used for the selection of the best-fitting model of sequence evolution for ITS dataset (GTR+I+G+F) and the combined dataset (GTR+I+G for ITS and nrLSU, SYM+I+G for RPB2), respectively. Both of the two data sets were run for 5 000 000 generations, with four chains, and trees sampled every 500 generations. The average split frequencies were checked to determine optimal convergence of the chains below 0.01. The first 25% of the sample trees was designated as burn-in, and the remaining samples were retained for further analyses. The topologies were used to generate a 50% majority-rule consensus tree for posterior probabilities (PP). The best tree was viewed in FIGTREE v1.4.4 (Rambaut 2018) and was compiled in Adobe Illustrator CC. Both of the final alignments were submitted to TreeBASE (Submission ID 28200).

## Results

### Molecular phylogenetic results

**Figure 1.**
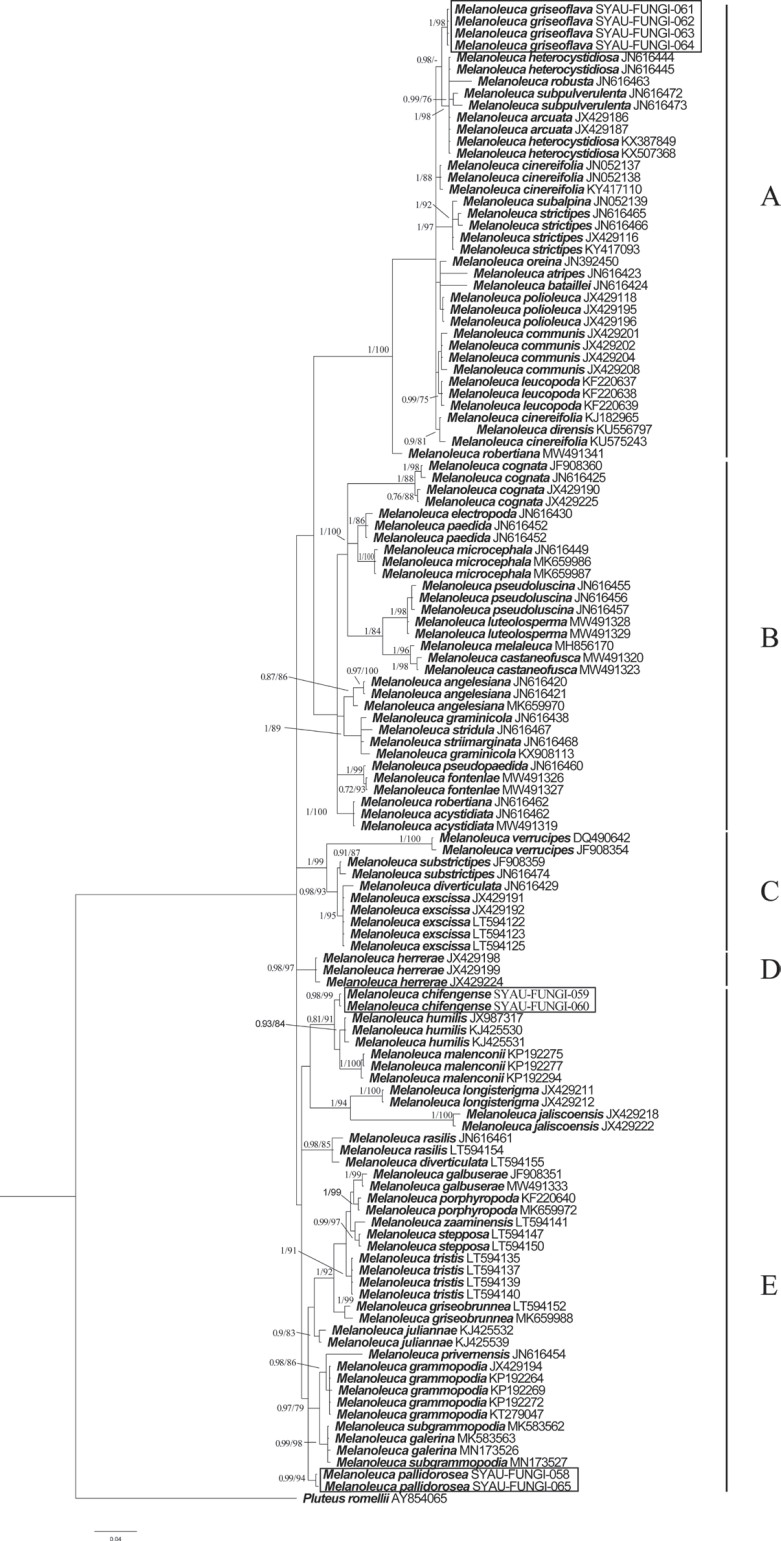
Phylogenetic placements of the three new *Melanoleuca*, inferred from the ITS region using MrBayes. The lineages with new species were shown in boxes. PP ≥ 0.95 and BS ≥ 75% were indicated around the branches. Accession numbers of ITS in GenBank follow the fungal names.

**Figure 2.**
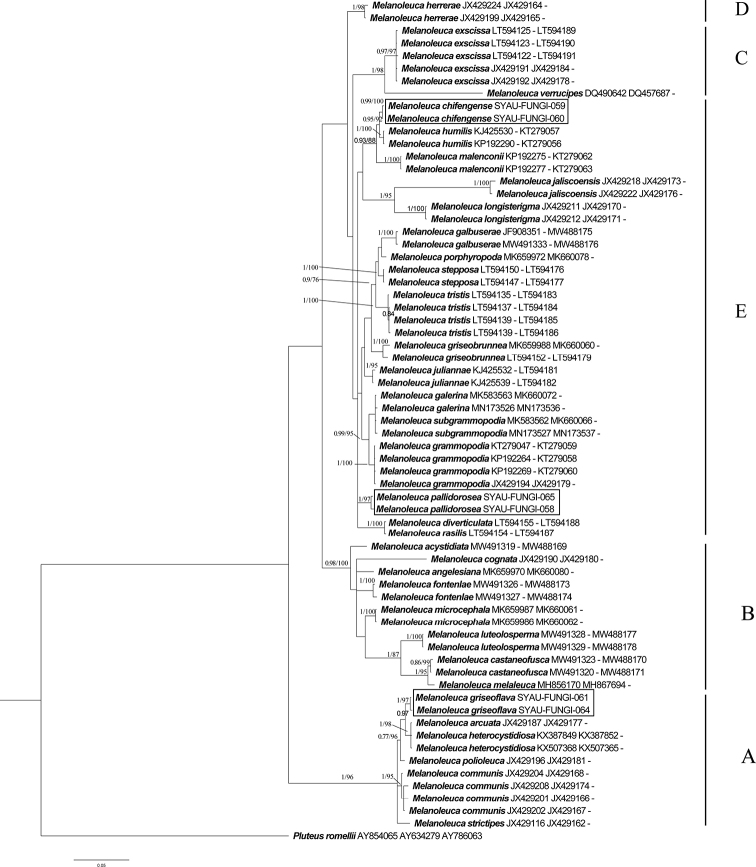
Phylogenetic placements of the three new *Melanoleuca*, inferred from the combined regions (ITS-nrLSU-RPB2) using MrBayes. The lineages with new species were shown in boxes. PP ≥ 0.95 and BS ≥ 75% were indicated around the branches. Accession numbers in GenBank (ITS, nrLSU, RPB2) follow the fungal names.

The GenBank accession numbers of the sequences, determined in this study, are from MW258676 to MW258689 and MW281543 to MW281548 (Table 1). The BI and ML analyses produced similar topologies for the ITS and combined regions datasets. The BI trees were selected for display (Figures 1, 2). The results showed that the species in the genus *Melanoleuca* formed a monophyletic group in both ITS regions and combined regions analyses (PP=1.00, BS=100, Figures 1, 2), which is consistent with the previous results (Yu et al. 2014; Vizzini et al. 2011). A total of five clades (A to E) can be recognized within *Melanoleuca* (Figures 1, 2). Based on the analyses of the two datasets, the collections named *M. grisoflava* (SYAU-FUNGI-061 to SYAU-FUNGI-064) formed an independent lineage with strong statistical support (PP = 1.00, BS ≥ 97), located within clade A, and sister to a clade containing sequences of *M. arcuata* (Bull.) Singer, *M. heterocystidiosa* (Beller & Bon) Bon, *M. robusta* (Bres.) Fontenla, Gottardi & Para and *M. subpulverulenta* (Pers.) Singer. In clade E, *Melanoleuca chifengense* consist of two collections (SYAU-FUNGI-059 and SYAU-FUNGI-060) that form an independent lineage with high support (PP≥0.98, BP≥99) and close to *M. humilis* (Pers.) Pat. and *M. malenconii* Bon. The collections (SYAU-FUNGI-058 and SYAU-FUNGI-065) named *M. pallidorosea* group together in clade E with well support (PP≥0.99, BP≥94).

**Table 1.** Collections of *Melanoleuca* used for DNA sequence analyses.

Species	Voucher collection	Origin	GenBank accession No.		
			ITS	nrLSU	RPB2
*Melanoleuca pallidorosea*	SYAU-FUNGI-058	Xilingole League, Inner Mongolia, China	MW258676	MW258684	MW281543
*M. pallidorosea*	SYAU-FUNGI-065	Xilingole League, Inner Mongolia, China	MW258677	MW258687	MW281545
*M. griseoflava*	SYAU-FUNGI-061	Fuxin City, Liaoning Province, China	MW258680	MW258685	MW281544
*M. griseoflava*	SYAU-FUNGI-062	Shenyang City, Liaoning Province, China	MW258681	–	–
*M. griseoflava*	SYAU-FUNGI-063	Shenyang City, Liaoning Province, China	MW258682	–	–
*M. griseoflava*	SYAU-FUNGI-064	Chifeng City, Inner Mongolia, China	MW258683	MW258686	MW281548
*M. chifengense*	SYAU-FUNGI-059	Chifeng City, Inner Mongolia, China	MW258678	MW258688	MW281546
*M. chifengense*	SYAU-FUNGI-060	Chifeng City, Inner Mongolia, China	MW258679	MW258689	MW281547

### Taxonomy

#### Melanoleuca chifengense

Taxon classificationFungiAgaricalesTricholomataceae

X.D. Yu & H.B. Guo sp. nov.

4E61A08B-98F0-54D7-9811-8A73DD214E82

838026

Figs 3 , 6a–c

##### Etymology.

The epithet refers to the species found in Chifeng City in north-eastern China.

##### Diagnosis.

The new species is distinguished from *M. exscissa* in having yellowish tinct pileus and without any type of cystidia.

##### Type.

China. Inner Mongolia: Chifeng City, Linxi County, Xinlin Town, Dauran Village, alt. 1200 m, 44.00°N, 118.07°E, 21 Aug 2017, H.B. Guo (SYAU-FUNGI-059).

**Figure 3.**
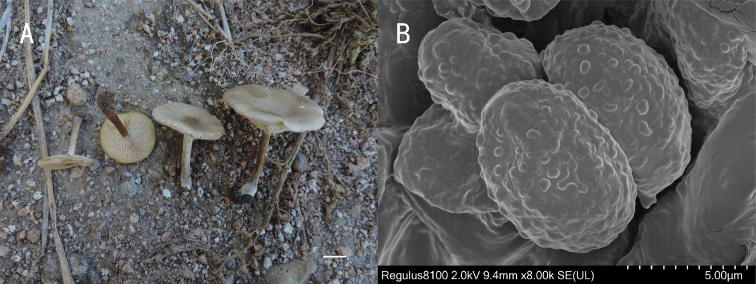
*Melanoleuca chifengense* (holotype, SYAU-FUNGI-059) **A** macroscopic habit **B** surface of basidiospores. Scale bars: 1 cm (**A**); 5 μm (**B**).

##### Description.

Pileus 30–60 mm diam., flat at first, becoming depressed at disc when mature, margin sometimes cracking, surface glabrous, grey to yellowish-grey (4B1 to 4B2), greyish-brown (4B4 to 4B6) at centre, often darker at margin. Lamellae crowded, adnate to decurrent, white to yellowish-white (4A2), 2.5–3.0 mm broad, with lamellulae, edge entire. Stipe cylindrical, 20–35 mm long × 2–5 mm diam., central, broadened at base, solid, surface grey to yellowish-grey at first (4B1 to 4B2), becoming yellowish-brown (5D8, 5E8) with age or after touching, striate, often with whitish basal tomentum. Pileus context up to 10 mm thick near stipe attachment, thin at margin, yellowish-grey (4B2), grayish brown to yellowish brown (5D3 to 5E5) in stipe cortex, up to brown (6E7) in stipe base. Odour none, taste mild. Spore print white.

Basidiospores (90/6/2) 7.0–8.5 (9.0) × 4.0–6.2(6.5) μm, av. 7.5 × 5.2 μm, Q = 1.40–1.45(1.50), ellipsoid, hyaline, amyloid, ornamentation verruculose, with irregular warts, sometimes with ridges. Basidia (20) 23–29 (30) × (7.0) 7.5–9.0 (10.0) μm, av. 26 × 8.5 μm, clavate, 4-spored, sometimes 2-spored, subhyaline. Hymenial cystidia absent, lamella edge sterile. Hymenophoral trama 42–85 μm broad, regular with thin-walled hyphae 5.5–16.5 μm diam., hyphae not pigmented. Subhymenium poorly developed. Pileipellis a cutis of numerous repent branched hyphae, 5.5–7.5 μm wide, thin-walled. Stipitipellis hyphae 3.5–8.0 μm diam., thin-walled, hyaline. Caulocystidia absent. Clamp connections absent.

##### Habit, ecology and distribution.

On soil or meadow outside of a forest, often on the roadside near a forest. Known from north-eastern China.

##### Additional specimens examined.

China. Inner Mongolia: Chifeng City, Linxi County, Xinlin Town, Dauran Village, alt. 1201 m, 44.07°N, 118.08°E, 22 Aug 2017, H.B. Guo (SYAU-FUNGI-060).

#### Melanoleuca griseoflava

Taxon classificationFungiAgaricalesTricholomataceae

X.D. Yu & H.B. Guo sp. nov.

E6EA1538-5438-5C39-845B-897F66F8EA13

838027

Figs 4 , 6d–g

##### Etymology.

The epithet refers to the colour of the pileus which is greyish-brown.

##### Diagnosis.

The new species is distinguished from *M. exscissa* in having adnexed to adnate lamellae and fusiform cheilocystidia.

##### Type.

China. Liaoning Province: Shenyang City, Tianzhu Mountain, on the soil in woods, 31 Aug 2019, X.D. Yu (holotype: SYAU-FUNGI-062).

**Figure 4.**
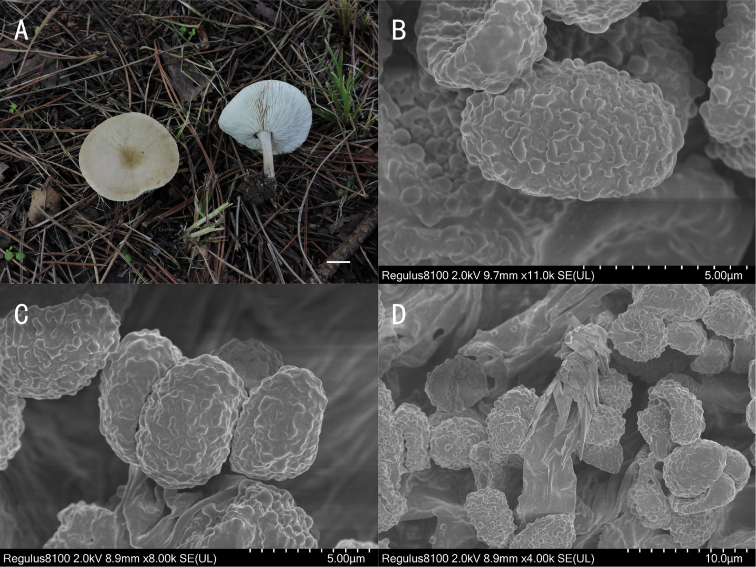
*Melanoleuca griseoflava* (holotype, SYAU-FUNGI-062) **A** macroscopic habit **B, C** surface of basidiospores **D** hymenial cystidia with encrusted crystals at apex. Scale bars: 1 cm (**A**); 5 μm (**B, C**); 10 μm (**D**).

##### Description.

Pileus 35–60 mm diam., flat at first, then gradually depressed, margin slightly inflexed when mature, surface fibrillose, greyish-brown (4B3 to 4B5), becoming deep yellow (4C6 to 4C8) at centre. Lamellae crowded, adnexed to adnate, white, 2.5–3.0 mm broad, with lamellulae, edge entire. Stipe cylindrical, 30–50 mm long × 3–5 mm diam., central, somewhat broadened at the base, fibrous, expanded at base, solid, surface yellowish-grey to greyish at first (4B2 to 4C2), becoming yellowish-brown (5E7 to 5E8) with age, striate, with whitish basal tomentum. Pileus context up to 10 mm thick near stipe attachment, thin at margin, greyish-yellow to yellowish-grey (4B4 to 4B2), yellowish-grey (4B2) in stipe cortex, whitish in stipe base. Odour none, taste mild. Spore deposit white.

Basidiospores (234/10/8) (5.0) 6.0–7.2 (8.0) × 4.0–5.0 (6.0) μm, av. 6.5 × 4.5 μm, Q = (1.30)1.45–1.55 (1.60), ellipsoid, hyaline, amyloid, ornamentation verruculose, warts with ridges, almost reticulate. Basidia (18) 20–25 (28) × (4.0) 5.0–6.5 (7.0) μm, av. 22 × 6.0 μm, clavate, 4-spored, occasionally 2-spored, hyaline. Cheilocystidia (40) 45–55 (60) × (6.0) 8.0–12.0 (15.0) μm, fusiform, thin-walled, with encrusted crystals at apex, abundance. Pleurocystidia scattered, similar to cheilocystidia. Hymenophoral trama 90–150 μm broad, regular with thin-walled hyphae 3.0–14.0 μm diam., hyphae not pigmented, lamella edge sterile, Subhymenium poorly developed. Pileipellis a cutis of numerous repent branched hyphae 7.5–10.5 μm wide, thin-walled, pigmented with light violet. Stipitipellis hyphae 3–10.0 μm diam., smooth, thin-walled, pigmented. Caulocystidia of two types of cells, (1) 40–90 × 6.0–10.0 μm, fusiform, thin-walled, some with encrusted crystals at apex, similar to cheilocystidia; (2) 30–40 × 7.0–10.0 μm, clavate, thin-walled, without crystals. Clamp connections absent.

##### Habit, habitat and distribution.

Solitary, saprotrophic on the soil, on the grass, on roadsides, in woods. Known from north-eastern China.

##### Additional specimens examined.

China. Liaoning Province: Shenyang City, Tianzhu Mountain, on the soil in woods, 31 Aug 2019, X.D. Yu (SYAU-FUNGI-063); Fuxin City, Haitang Mountain, on roadsides, 20 Jul 2019, H.B. Guo (SYAU-FUNGI-061). Inner Mongolia: Chifeng City, Linxi County, Xinlin Town, Dauran Village, alt. 1286 m, 43.08°N, 118.07°E, 22 Aug 2017, X.D. Yu (SYAU-FUNGI-064). Chifeng City, Karakqin Banner, Maanshan, 2 Sep 2019, T.Z. Liu & Y.M. Gao (CFSZ 21439).

#### Melanoleuca pallidorosea

Taxon classificationFungiAgaricalesTricholomataceae

X.D. Yu & H.B. Guo sp. nov.

BAA9BA5B-CBA5-5AB1-8F35-16252AC25C77

838028

Figs 5 , 6h, i

##### Etymology.

The epithet refers to the species which has a pallid rose pileus.

##### Diagnosis.

The new species is distinguished from *M. grammopodia* and *M. leucopoda* in having a pinkish-white pileus.

##### Type.

China. Inner Mongolia: Xilingole League, Xiwuzhumuqin Banner, on the grass in woods, 1051 m alt., 44.48°N, 117.86°E, 22 Aug 2017, X.D. Yu (holotype: SYAU-FUNGI-058).

**Figure 5.**
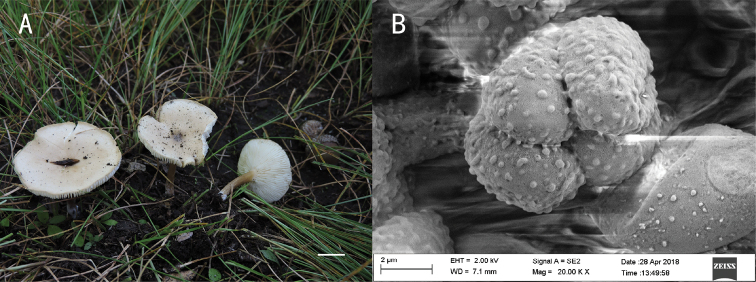
*Melanoleuca pallidorosea* (holotype, SYAU-FUNGI-058) **A** macroscopic habit **B** surface of basidiospores. Scale bars: 1 cm (**A**); 2 μm (**B**).

##### Description.

Pileus 30–65 mm diam., flat, with depressed centre, margin slightly undulating, expanding to uplifted, sometimes slightly lacerate when mature, surface glabrous, camel (9E8 to 10E8) at centre, pinkish-white (10A3 to 10A4) towards the margin. Lamellae rather distant, adnate to decurrent, white, 3.0–4.5 mm broad, with lamellulae of two lengths, but not intervening, edge entire. Stipe cylindrical, 20–50 mm long × 5–8 mm diam., in upper part of stipe apricot (6C8 to 6D8), becoming yellowish-brown (5E8) towards base, with whitish flocculose apex, longitudinally striate, with whitish basal tomentum. Context up to 2–5 mm thick at the pileus base, whitish to creamy, whitish in stipe cortex and base. Smell fungoid smell, taste mild. Spore print white.

Basidiospores (130/7/4) (6.5) 7.0–8.5 (9.0) × 5.0–6.0 (6.5) μm, av. 7.4 × 5.5 μm, Q = (1.28)1.31–1.40(1.44), ellipsoid, hyaline, ornamentation verruculose, warts mainly round and scattered, amyloid. Basidia (20) 25–33 (35) × (6.0) 6.5–9.5 (10.5) μm, av. 28 × 8.5 μm, clavate, 4-spored, occasionally 2-spored, subhyaline. Hymenial cystidia absent. Lamella edge sterile. Hymenophoral trama 95–159 μm wide, regular, with thin-walled hyphae, 5.0–10.0 μm diam., hyphae not pigmented. Subhymenium poorly developed. Pileipellis a cutis of numerous repent branched hyphae, 4.0–10.0 μm wide, inflated cell to 21.0 μm, thin-walled. Stipitipellis hyphae 7.0–10.0 μm, thin-walled, hyaline. Caulocystidia absent. Clamp connections absent.

##### Habit, ecology and distribution.

Solitary or in small group, saprotrophic in grass. Known from north-eastern China.

##### Additional specimens examined.

China. Inner Mongolia: Xilingole League, Xiwuzhumuqin Banner, on the grass in woods, 1051 m alt., 44.48°N, 117.86°E, 22 Aug 2017, X.D. Yu (SYAU-FUNGI-065). Chifeng City, Bahrain Banner Saihanwula, 10 Sep 2016, T.Z. Liu & Z.L. Song (CFSZ 12136); 12 Sep 2016, T.Z. Liu & Z.L. Song (CFSZ 12253).

**Figure 6.**
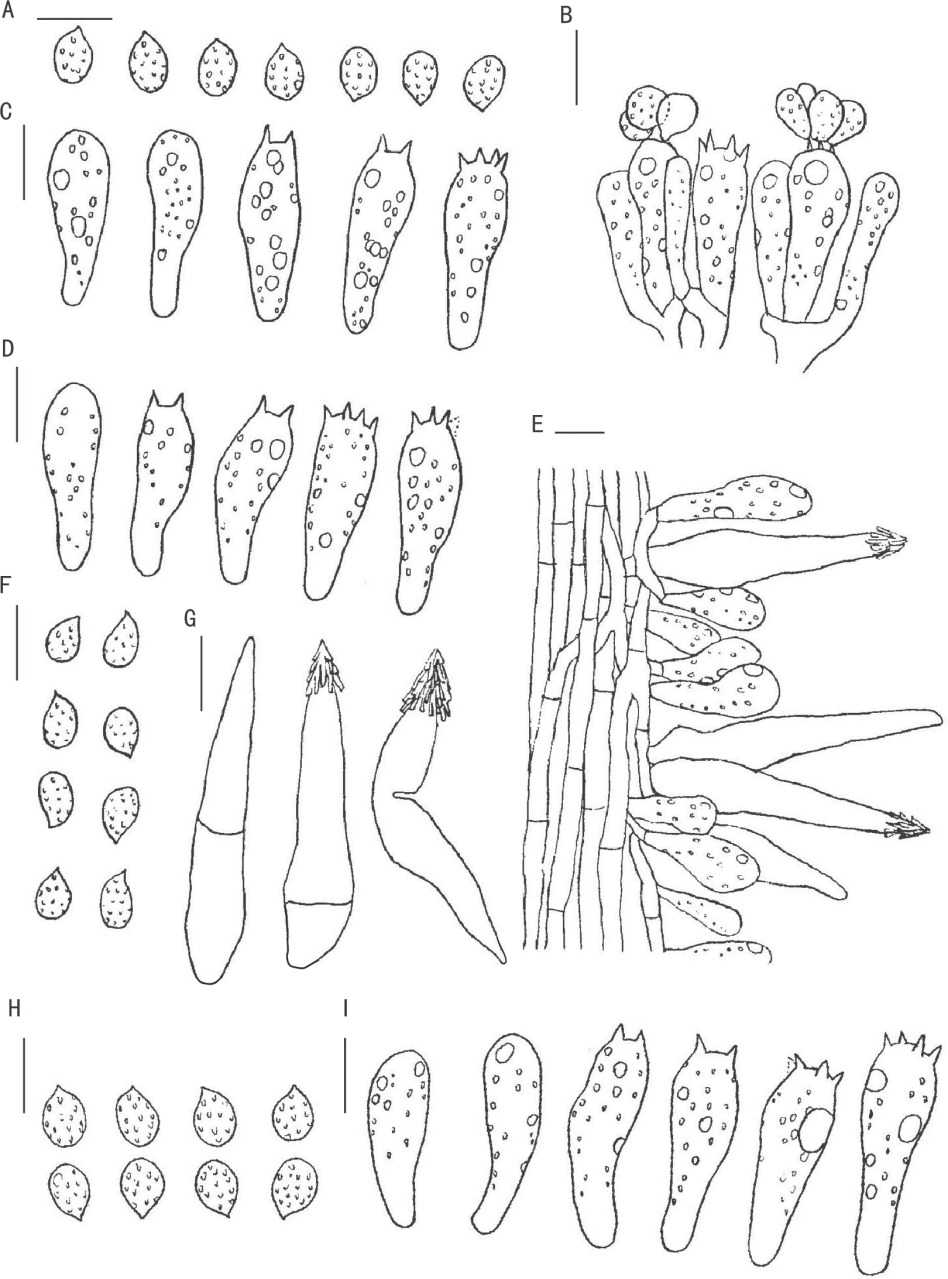
Line drawings of the three new *Melanoleuca* species **A–C***Melanoleuca chifengense* (holotype, SYAU-FUNGI-059) **A** basidiospores **B, C** basidia and basidioles **D–G***Melanoleuca griseoflava* (holotype, SYAU-FUNGI-062) **D** basidia and basidioles **E** caulocystidia **F** basidiospores **G** cheilocystidia **H, I***Melanoleuca pallidorosea* (holotype, SYAU-FUNGI-058) **H** basidiospores **I** basidia and basidioles. Scale bars: 10 μm.

## Discussion

Morphologically, the most distinctive features of *M. pallidorosea* are a pinkish-white pileus, a yellowish stipe, white and decurrent lamellae, lack of hymenial cystidia, ellipsoid basidiospores with round and scattered warts, 7.0–8.5 × 5.0–6.0 μm. According to the classification system of Singer (1986), *Melanoleuca pallidorosea* should belong to sect. Alboflavidae because of the pinkish-white pileus. Four species with a whitish pileus in the section were similar to *M. pallidorosea*, i.e. *M. balansae* (Speg.) Singer (Spegazzini 1883), *M. candida* Singer (Singer 1943), *M. kavinae* (Pilát & Veselý) Singer (Pilát and Veselý 1932) and *M. strictipes* (P. Karst.) Jul. Schäff. (Ďuriška et al. 2017). The latter three species mainly differ on account of their large pileus size (up to 12 cm diam.). Moreover, all of them have macrocystidia which differs from *M. pallidorosea*. Considering the size of the pileus (up to 6 cm diam.), *M. balansae* (Speg.) Singer is similar to *M. pallidorosea* to some extent. However, *M. balansae*, originally reported from Paraguay, differs on account of its white stipe and smaller basidiospores (7–7.5 × 4–5 μm).

*Melanoleuca chifengense* is easily recognised by its grey to yellowish-grey pileus, decurrent lamellae, grey to yellowish-brown stipe and yellowish-grey context, and lack of hymenial cystidia. *Melanoleuca griseoflava* is characterised by a greyish-brown pileus, adnexed to adnate lamellae, yellowish-grey stipe, greyish-yellow context and fusiform cystidia. The two species have similar-sized basidiomata and grey pileus, *Melanoleuca griseoflava* differs from *M. chifengense* by the adnexed to adnate gills and having abundant fusiform cystidia. According to Singer (1986), both *M. chifengense* and *M. griseoflava* belonged to sect. Oreinae, based on their grey pileus, narrow lamellae and nearly pallid stipe. Amongst the section Oreinae, the two new species differ from the other species by their small-size basidiomata, including *M. catalaunica* Singer, *M. graminicola* (Velen.) Kühner & Maire, *M. microcephala* (P. Karst.) Singer and *M. oreina* (Fr.) Kühner & Maire (Singer 1943). Some species in sect. Oreinae have the urticoid hymenial cystidia, making them easily distinguishable from *M. chifengense* and *M. griseoflava*, such as *M. paedida* (Fr.) Kühner & Maire (Vizzini et al. 2011), *M. exscissa* (Fr.) Singer (Antonín et al. 2017), *M. humilis* (Pers.) Pat. (Antonín et al. 2015), and *M. rasilis* (Fr.) Singer (Antonín et al. 2017). *Melanoleuca griseoflava* can be distinguished from the above species by its fusiform hymenial cystidia. In addition, *M. chifengense* differs from them by its lack of any form of cystidia; *Melanoleuca subcinereiforme* Murrill, originally reported in Oregon, differs on account of its finely pruinose, smoky pileus and white stipe (Murrill 1914); *Melanoleuca deserticola* (Speg.) Singer mainly differs on account of its spotted-pileus, short and solid stipe and larger basidiospores (9–11 × 4–6 μm) (Spegazzini 1900); *M. strictipes* differs by its larger basidiomata (55–115 mm broad), leathery yellow pileus and a distinct bulb stipe (Ďuriška et al. 2017).

In the present study, both phylogenetic analyses, based on a single region (ITS) and three regions (ITS-nrLSU-RPB2), showed that there were nine clades in the genus *Melanoleuca* (Figures 1, 2). According to the phylogram, *M. griseoflava* is sister to the other four species in clade A, i.e. *M. arcuata*, *M. heterocystidiosa*, *M. robusta* and *M. subpulverulenta*. *Melanoleuca arcuata* differs by its brick-red pileus and decurrent lamellae (Fries 1821). The other two species, *M. heterocystidiosa* and *M. subpulverulenta*, can also be easily separated from *M. griseoflava*, based on their small basidiomata (Singer 1939; Bon 1984); *M. robusta* differs on account of its grey-brown pileus, grey lamellae, brown context and caespitose growth (Vizzini et al. 2011). In clade E, *M. chifengense* is closely related to *M. humilis* and *M. malenconii* with high support. However, both the two species differ from *M. chifengense* in their dark brown pileus (Fries 1821; Bon 1990). In the analysis of both ITS region and three regions (ITS-nrLSU-RPB2), *Melanoleuca pallidorosea* form an individual clade (clade I) and far away from the other species of *Melanoleuca*.

## Supplementary Material

XML Treatment for Melanoleuca chifengense

XML Treatment for Melanoleuca griseoflava

XML Treatment for Melanoleuca pallidorosea
